# Association of lipoprotein indices with 3-month functional outcome after ischemic stroke: a retrospective cohort study

**DOI:** 10.3389/fnut.2026.1826387

**Published:** 2026-08-03

**Authors:** Zichen Rao, Chunyi Xu, Yanmei Yu, Genying Zhu, Yong Liu, Xuehua Wen, Chunyan Zhu, Yiming Zhang

**Affiliations:** 1 Department of Endocrinology, Quzhou People’s Hospital, The Quzhou Affiliated Hospital, Wenzhou Medical University, Quzhou, Zhejiang, China; 2 Department of Endocrinology and Metabolism, Sir Run Run Shaw Hospital, Zhejiang University School of Medicine, Hangzhou, China; 3 Department of Rehabilitation Medicine, Center for Rehabilitation Medicine, Rehabilitation & Sports Medicine Research Institute of Zhejiang Province, Zhejiang Provincial People's Hospital (Affiliated People's Hospital Hangzhou Medical College), Hangzhou, Zhejiang, China; 4 Department of Radiology, Center for Rehabilitation Medicine, Zhejiang Provincial People's Hospital (Affiliated People's Hospital Hangzhou Medical College), Hangzhou, Zhejiang, China

**Keywords:** acute ischemic stroke, atrial fibrillation, functional outcome, LDL-related ratios, lipoprotein ratios, low-density lipoprotein cholesterol

## Abstract

**Background:**

Lipid-related parameters have been linked to vascular risk, yet their association with post-stroke functional recovery remains uncertain. We aimed to examine whether lipoprotein imbalance is associated with 3-month functional outcome after acute ischemic stroke.

**Methods:**

In this retrospective cohort study, 748 patients with acute ischemic stroke were included. Poor functional outcome was defined as modified Rankin Scale (mRS) > 2 at 3 months. Fourteen lipid-related parameters were analyzed. Multivariable logistic regression, generalized additive models, two-piecewise regression, and subgroup analyses were performed with adjustment for age, sex, smoking, hypertension, diabetes, atrial fibrillation (AF), Trial of Org 10,172 in Acute Stroke Treatment (TOAST) classification, and baseline National Institutes of Health Stroke Scale (NIHSS) score.

**Results:**

LDL-related measures were independently associated with poor functional outcome. Specifically, each unit increase in lbLDL-C/HDL-C (OR 1.86, 95% CI 1.32–2.63, *p* < 0.001) and TC/HDL-C (OR 1.27, 95% CI 1.02–1.57, *p* = 0.031) was linked to higher risk of poor functional outcome. Compared with the lowest quartile, patients in the highest quartile of lbLDL-C/HDL-C (OR 3.10, 95% CI 1.60–6.01, *p* < 0.001) and LDL-C/HDL-C (OR 2.85, 95% CI 1.43–5.67, *p* = 0.003) showed the strongest associations. Most LDL-related indices including NHHR, TC, and lbLDL-C showed positive associations (all *p* < 0.05), whereas TG and TG/HDL-C displayed decreasing trends. Exploratory smooth-curve and threshold analyses suggested non-linear patterns for HDL-C and remnant cholesterol, although remnant cholesterol was not independently associated with functional outcome in the primary adjusted analyses. AF significantly modified the associations of lipid ratios with outcome, with stronger associations observed among patients without AF.

**Conclusion:**

Lipoprotein imbalance, reflected by LDL-related ratios, was associated with 3-month functional outcome after ischemic stroke. The observed non-linear patterns and interaction with AF suggest heterogeneity in lipid-outcome associations after ischemic stroke. These exploratory findings require further validation in independent cohorts.

## Introduction

Acute ischemic stroke remains a leading cause of long-term disability worldwide (1). Although advances in acute management have improved survival, functional recovery after stroke varies substantially among individuals (2). Established predictors such as age and baseline stroke severity explain part of this variability, yet considerable heterogeneity remains unexplained (3).

Lipid abnormalities are well recognized as contributors to atherosclerosis and vascular risk (4). Conventional lipid measures, particularly low-density lipoprotein cholesterol (LDL-C), have been extensively studied in relation to primary and secondary stroke prevention (5). However, the relationship between lipid profiles and post-stroke functional outcome is less clear. Prior studies have yielded inconsistent findings, and most have focused on single lipid concentrations rather than on indices reflecting the balance between atherogenic and protective lipoproteins.

Recent research indicates that lipid ratios and particle metrics more accurately reflect the balance between atherogenic burden and the protective functions of high-density lipoproteins (6). Mechanistically, lipoprotein imbalance may impede post-stroke recovery by exacerbating systemic inflammatory responses and endothelial dysfunction, both of which are detrimental to the structural integrity and functional remodeling of the neurovascular unit (7, 8). Furthermore, associations between lipid profiles and clinical outcomes frequently deviate from linear trajectories, and the potential for stroke mechanisms such as atrial fibrillation to modify these effects has seldom been investigated.

Therefore, we aimed to systematically evaluate a broad range of lipid-related parameters in relation to 3-month functional outcome after acute ischemic stroke. We assessed linear and non-linear associations, explored potential threshold effects, and examined interaction with key clinical characteristics, with particular attention to AF status.

## Methods

### Study design and participants

This retrospective cohort study included a consecutive inception cohort of patients with acute ischemic stroke admitted to our institution between 2021–2025. To ensure a well-defined inception point, we specifically recruited patients at a uniform early stage of the disease, defined as admission within 24 h of symptom onset. Acute ischemic stroke was diagnosed according to World Health Organization criteria and confirmed by magnetic resonance imaging.

Inclusion criteria were: (1) age ≥18 years; (2) admission within 24 h of symptom onset; (3) availability of complete lipid profile and 3-month functional outcome data.

After the initial screening and the exclusion of patients with documented active infection or missing 3-month mRS follow-up data, a total of 776 patients were identified. Active infection was operationally defined based on clinical diagnoses recorded in the medical records, supported by objective findings including: (1) body temperature >37.5 °C or <36.0 °C; (2) abnormal white blood cell count (>10 × 10^9^/L or <4 × 10^9^/L); or (3) radiological or microbiological evidence of infection (e.g., pulmonary infiltrates on chest imaging or positive cultures). All such patients were excluded to minimize potential confounding effects of acute inflammatory states on lipid profiles and functional outcomes. Furthermore, patients with severe hepatic or renal dysfunction (e.g., end-stage organ failure) or active malignancies were excluded to eliminate potential confounding effects on lipid metabolism and stroke recovery. Among the remaining 776 eligible patients, 28 (3.6%) were further excluded due to incomplete baseline lipid profiles, primarily resulting from emergency clinical interventions or early inter-departmental transfers that prevented blood sample collection within the required fasting window. Following STROBE recommendations for addressing potential selection bias, a complete case analysis was employed without statistical imputation. Since the proportion of missing data was minimal (3.6%) and considered missing at random due to administrative factors rather than clinical severity, this approach is unlikely to significantly bias the effect estimates. Ultimately, 748 patients with complete clinical and laboratory data were included in the final analysis. The study protocol was approved by the institutional ethics committee, and the requirement for informed consent was waived due to the retrospective design.

### Data collection

Baseline demographic and clinical characteristics were obtained from the hospital information system, including age, sex, smoking status, medical history (hypertension, diabetes, atrial fibrillation), Trial of Org 10,172 in Acute Stroke Treatment (TOAST) classification, and baseline stroke severity assessed by the National Institutes of Health Stroke Scale (NIHSS).

According to the standardized clinical protocol of our institution, fasting blood samples were routinely collected on the first morning after admission, following an overnight fast of at least 8 h. Laboratory measurements included total cholesterol (TC), triglycerides (TG), high-density lipoprotein cholesterol (HDL-C), low-density lipoprotein cholesterol (LDL-C), and related indices.

Large buoyant LDL-C (lbLDL-C) was calculated using the validated Sampson formula (9):


$$\begin{array}{l} \text{lbLDL}-C\;(\mathrm{mg}/\mathrm{dL})=1.43\times \mathrm{LDL}-C-0.14\\ \times [\ln\;(\mathrm{TG})\times \mathrm{LDL}-C]-8.99 \end{array}$$

Small dense LDL-C (sdLDL-C) was derived as LDL-C − lbLDL-C.

Remnant cholesterol (RC) was calculated as TC − LDL-C − HDL-C.

Non–HDL-C to HDL-C ratio (NHHR) was calculated as (TC − HDL-C)/HDL-C.

The primary outcome was poor functional outcome at 3 months, defined as modified Rankin Scale (mRS) > 2.

### Statistical analysis

Statistical Power and Sample Size: The sample size was determined by the number of consecutive patients meeting the eligibility criteria during the study period. A total of 748 patients were ultimately included. Based on the observed incidence of unfavorable functional outcomes (26.3%) in our cohort, this sample size provides an adequate event-per-variable (EPV) ratio for the multivariable logistic regression models, ensuring the stability and reliability of the estimated associations.

Statistical analyses were performed using EmpowerStats (version 2.0) and R software. A two-sided *p* value <0.05 was considered statistically significant.

Continuous variables were assessed for normality using the Shapiro–Wilk test. Normally distributed variables are presented as mean ± standard deviation and compared using Student’s t-test. Non-normally distributed variables are presented as median (interquartile range) and compared using the Mann–Whitney U test. Categorical variables are presented as counts (percentages) and compared using the chi-square test.

Multivariable logistic regression models were constructed to evaluate associations between lipid-related parameters and poor 3-month functional outcome. Potential confounders were selected based on their established clinical significance in stroke prognosis and prior literature. Three models were developed:

Model 1: unadjusted;
Model 2: adjusted for age and sex;
Model 3 (fully adjusted): additionally adjusted for smoking status, hypertension, diabetes, atrial fibrillation, TOAST classification, and baseline NIHSS score to minimize residual confounding.

Lipid parameters were analyzed both as continuous variables and as quartiles, with tests for trend performed across quartiles.

To explore potential non-linear associations, generalized additive models (GAMs) with smoothing splines were applied under the fully adjusted framework.

For variables showing potential non-linearity, two-piecewise logistic regression models were used to identify inflection points. Log-likelihood ratio tests were performed to compare the piecewise model with the linear model.

Sensitivity analyses were performed by excluding extreme outliers for core indices (lbLDL-C/HDL-C and NHHR), defined as values exceeding 3 standard deviations from the cohort mean. To further account for inflammatory burden, an additional sensitivity analysis was performed by adding C-reactive protein to the fully adjusted model. This CRP-adjusted analysis focused on the principal LDL-related lipid ratios that showed independent associations in the primary analyses, including lbLDL-C/HDL-C, LDL-C/HDL-C, TC/HDL-C, and NHHR. To assess whether these LDL-related lipid ratios provided incremental prognostic information beyond established clinical predictors, we further compared a clinical base model with extended models that separately added each lipid ratio. The clinical base model included age, sex, smoking status, hypertension, diabetes, atrial fibrillation, TOAST classification, and baseline NIHSS score. Model discrimination was assessed using the area under the receiver operating characteristic curve (AUC). AUC confidence intervals and *p* values for AUC comparison were estimated using nonparametric bootstrap resampling with 500 repetitions. Net reclassification improvement (NRI) and integrated discrimination improvement (IDI) were calculated to evaluate reclassification and discrimination improvement after adding each lipid ratio.

Subgroup analyses were conducted to examine potential effect modification by age, sex, smoking status, hypertension, diabetes, atrial fibrillation, and TOAST classification. Interaction terms were tested in the fully adjusted model.

## Results

### Baseline characteristics of the study population

A total of 748 patients were included, of whom 551 (73.7%) achieved favorable functional outcome (mRS ≤ 2) and 197 (26.3%) had poor functional outcome at 3 months. Patients with poor functional outcome were older and more frequently had diabetes and atrial fibrillation. Sex distribution and smoking status also differed between groups. Baseline blood pressure, hypertension prevalence, and TOAST classification were comparable.

Regarding laboratory findings, the poor functional outcome group had higher fasting plasma glucose, HbA1c, C-reactive protein, homocysteine, white blood cell count, and lbLDL-C levels, while free triiodothyronine levels were lower. Traditional lipid parameters, including TC, LDL-C, HDL-C, and RC, did not differ significantly between groups.

Stroke severity markers differed substantially. Patients with poor functional outcome had higher baseline NIHSS, A2DS2, and KWDT scores and lower GCS scores (see Table 1).

**Table 1:** Baseline demographic, laboratory, and clinical characteristics according to 3-month functional outcome.

Demographic characteristic	3-month mRS ≤ 2	3-month mRS > 2	*p* value
*N*	551	197	
Age (years)	67.88 ± 11.65	73.48 ± 12.65	**<0.001**
SBP (mmHg)	153.00 (138.00–169.00)	156.00 (140.00–172.00)	0.117
DBP (mmHg)	83.00 (74.50–90.00)	82.00 (73.00–91.00)	0.970
Sex, *n* (%)			**0.002**
Female	341 (61.89%)	97 (49.24%)	
Male	210 (38.11%)	100 (50.76%)	
Smoking (*n*%)			**0.002**
No	344 (62.43%)	147 (74.62%)	
Yes	207 (37.57%)	50 (25.38%)	
Hypertension (*n*%)			0.345
No	127 (23.05%)	52 (26.40%)	
Yes	424 (76.95%)	145 (73.60%)	
Diabetes (*n*%)			**0.008**
No	374 (67.88%)	113 (57.36%)	
Yes	177 (32.12%)	84 (42.64%)	
AF (*n*%)			**<0.001**
No	481 (87.30%)	135 (68.53%)	
Yes	70 (12.70%)	62 (31.47%)	
TOAST (*n*%)			0.153
LAA	255 (49.51%)	81 (56.25%)	
Non-LAA	260 (50.49%)	63 (43.75%)	
Laboratory parameters			
FPG (mg/dL)	113.36 ± 44.28	123.92 ± 60.61	**<0.001**
HbA1c (%)	5.90 (5.60–7.10)	6.10 (5.50–7.30)	0.245
TG (mg/dL)	114.26 (79.71–165.63)	96.54 (70.86–131.97)	**<0.001**
TC (mg/dL)	164.73 (139.21–192.96)	169.76 (142.69–201.86)	0.393
HDL-C (mg/dL)	43.70 (37.51–51.04)	46.40 (38.67–53.36)	0.062
LDL-C (mg/dL)	104.41 (82.37–131.28)	110.60 (83.91–140.76)	0.214
RC (mg/dL)	13.53 (8.51–20.50)	13.15 (8.51–18.17)	0.196
lbLDL-C (mg/dL)	70.61 (56.02–86.63)	76.79 (57.50–98.02)	**0.019**
sdLDL-C (mg/dL)	33.51 (24.85–43.76)	32.25 (23.34–41.87)	0.147
TG/HDL-C	2.70 (1.70–4.10)	2.12 (1.45–3.41)	**<0.001**
TC/HDL-C	3.81 (3.09–4.57)	3.75 (3.11–4.45)	0.633
LDL-C/HDL-C	2.46 (1.82–3.13)	2.40 (1.89–3.11)	0.818
lbLDL-C/HDL-C	1.66 (1.22–2.10)	1.66 (1.26–2.22)	0.161
sdLDL-C/HDL-C	0.80 (0.54–1.06)	0.71 (0.52–1.01)	0.060
RC/HDL-C	0.31 (0.18–0.49)	0.28 (0.19–0.44)	0.145
NHHR	2.81 (2.09–3.57)	2.75 (2.11–3.45)	0.633
CRP (mg/L)	2.45 (1.20–5.50)	6.00 (2.00–20.00)	**<0.001**
AST (U/L)	19.00 (16.00–24.00)	20.20 (17.00–26.00)	**0.018**
ALT (U/L)	18.00 (13.00–27.00)	15.30 (12.00–23.00)	**0.028**
eGFR (ml/min/1.73 m^2^)	100.69 (80.58–121.56)	98.93 (76.87–118.81)	0.098
UA (umol/L)	320.90 (259.50–387.05)	319.90 (243.00–376.00)	0.432
HCY (umol/L)	15.00 (11.66–19.03)	16.60 (13.10–20.30)	**0.003**
WBC (*10^9^/L)	6.81 (5.42–8.31)	7.76 (6.29–9.90)	**<0.001**
FT3/FT4	0.30 (0.26–0.34)	0.26 (0.23–0.30)	**<0.001**
Clinical characteristics			
Baseline NIHSS	2.00 (1.00–4.00)	6.00 (3.00–10.00)	**<0.001**
KWDT	1.00 (1.00–1.00)	1.00 (1.00–3.00)	**<0.001**
GCS	15.00 (14.00–15.00)	13.00 (11.00–14.00)	**<0.001**
A2DS2	2.00 (2.00–3.00)	4.00 (3.00–5.00)	**<0.001**

Continuous variables are presented as mean ± standard deviation or median (interquartile range), as appropriate. Categorical variables are presented as number (percentage). Between-group comparisons were performed using Student’s t test or the Mann–Whitney U test for continuous variables and the chi-square test for categorical variables. A two-sided *p* value <0.05 was considered statistically significant. Statistically significant values are highlighted in bold. SBP, systolic blood pressure; DBP, diastolic blood pressure; AF, atrial fibrillation; TOAST, Trial of Org 10,172 in Acute Stroke Treatment classification; FPG, fasting plasma glucose; HbA1c, glycated hemoglobin; TG, triglycerides; TC, total cholesterol; HDL-C, high-density lipoprotein cholesterol; LDL-C, low-density lipoprotein cholesterol; lbLDL-C, large buoyant low-density lipoprotein cholesterol; sdLDL-C, small dense low-density lipoprotein cholesterol; RC, remnant cholesterol; NHHR, non–HDL-C to HDL-C ratio; CRP, C-reactive protein; AST, aspartate aminotransferase; ALT, alanine aminotransferase; eGFR, estimated glomerular filtration rate; UA, uric acid; HCY, homocysteine; WBC, white blood cell count; FT3, free triiodothyronine; FT4, free thyroxine; FT3/FT4, free triiodothyronine to free thyroxine ratio; NIHSS, National Institutes of Health Stroke Scale; GCS, Glasgow Coma Scale; A2DS2, Atrial fibrillation, Age, Dysphagia, Sex, and Stroke Severity score; KWDT, Kwong Wah Dysphagia Test.

### Multivariable associations between lipid parameters and 3-month functional outcome

In fully adjusted logistic regression models (Table 2), several lipid parameters were independently associated with poor functional outcome.

**Table 2:** Multivariable logistic regression analysis of the associations between lipid parameters and poor 3-month functional outcome.

Exposure	Crude model (Model 1)		Partially adjusted model (Model 2)		Fully adjusted model (Model 3)	
	OR (95% CI)	*p* value	OR (95% CI)	*p* value	OR (95% CI)	*p* value
(a)						
TG	1.00 (0.99, 1.00)	**0.0025**	1.00 (0.99, 1.00)	0.0577	1.00 (1.00, 1.00)	0.6033
TG quartile						
Q1	1.0		1.0		1.0	
Q2	0.71 (0.46, 1.10)	0.1264	0.80 (0.51, 1.26)	0.3393	1.34 (0.72, 2.48)	0.3557
Q3	0.56 (0.36, 0.88)	**0.0125**	0.64 (0.40, 1.02)	0.0585	0.95 (0.49, 1.83)	0.8730
Q4	0.48 (0.30, 0.76)	**0.0018**	0.63 (0.38, 1.03)	0.0644	1.11 (0.57, 2.19)	0.7580
P for trend	**0.0009**		**0.0348**		0.9498	
(b)						
TC	1.00 (1.00, 1.01)	0.3520	1.00 (1.00, 1.01)	0.3375	1.01 (1.00, 1.01)	**0.0255**
TC quartile						
Q1	1.0		1.0		1.0	
Q2	1.07 (0.67, 1.71)	0.7672	1.05 (0.65, 1.69)	0.8429	1.74 (0.88, 3.44)	0.1086
Q3	0.94 (0.58, 1.51)	0.7862	0.92 (0.57, 1.51)	0.7554	1.39 (0.71, 2.70)	0.3335
Q4	1.37 (0.87, 2.16)	0.1766	1.35 (0.84, 2.18)	0.2169	2.44 (1.27, 4.69)	**0.0072**
P for trend	0.2578		0.2967		**0.0171**	
(c)						
HDL-C	1.01 (1.00, 1.03)	0.1325	1.00 (0.98, 1.02)	0.9723	0.99 (0.97, 1.01)	0.5665
HDL-C quartile						
Q1	1.0		1.0		1.0	
Q2	1.05 (0.65, 1.71)	0.8365	0.98 (0.59, 1.60)	0.9243	1.06 (0.58, 1.96)	0.8414
Q3	1.27 (0.79, 2.03)	0.3237	1.10 (0.68, 1.79)	0.7007	1.14 (0.60, 2.14)	0.6960
Q4	1.56 (0.98, 2.49)	0.0600	1.14 (0.70, 1.87)	0.5898	0.91 (0.47, 1.73)	0.7650
P for trend	0.0394		0.4968		0.8163	
(d)						
LDL-C	1.00 (1.00, 1.01)	0.1928	1.00 (1.00, 1.01)	0.1222	1.01 (1.00, 1.02)	**0.0019**
LDL-C quartile						
Q1	1.0		1.0		1.0	
Q2	0.86 (0.53, 1.38)	0.5247	0.94 (0.58, 1.53)	0.8034	1.83 (0.92, 3.64)	0.0849
Q3	1.08 (0.68, 1.71)	0.7464	1.20 (0.75, 1.93)	0.4490	1.67 (0.84, 3.31)	0.1427
Q4	1.32 (0.84, 2.08)	0.2303	1.39 (0.87, 2.23)	0.1687	3.01 (1.55, 5.86)	**0.0012**
P for trend	0.1438		0.1059		**0.0022**	
(e)						
RC	0.99 (0.97, 1.00)	0.0408	0.99 (0.98, 1.00)	0.1238	0.99 (0.97, 1.00)	0.1385
RC quartile						
Q1	1.0		1.0		1.0	
Q2	1.16 (0.74, 1.83)	0.5154	1.16 (0.73, 1.85)	0.5316	1.16 (0.62, 2.17)	0.6342
Q3	1.21 (0.77, 1.90)	0.4185	1.28 (0.81, 2.04)	0.2912	1.45 (0.79, 2.67)	0.2339
Q4	0.69 (0.43, 1.13)	0.1418	0.76 (0.46, 1.26)	0.2934	1.03 (0.55, 1.96)	0.9170
P for trend	0.1963		0.4536		0.7303	
(f)						
lbLDL-C	1.01 (1.00, 1.01)	**0.0146**	1.01 (1.00, 1.01)	**0.0249**	1.01 (1.01, 1.02)	**0.0008**
lbLDL-C quartile						
Q1	1.0		1.0		1.0	
Q2	0.86 (0.53, 1.39)	0.5408	0.91 (0.56, 1.50)	0.7227	1.53 (0.79, 2.99)	0.2102
Q3	0.94 (0.59, 1.52)	0.8088	1.01 (0.62, 1.64)	0.9681	1.23 (0.63, 2.38)	0.5428
Q4	1.67 (1.07, 2.62)	**0.0245**	1.67 (1.05, 2.65)	**0.0294**	2.98 (1.58, 5.60)	**0.0007**
P for trend	**0.0197**		**0.0251**		**0.0018**	
(g)						
sdLDL-C	0.99 (0.98, 1.00)	0.2137	1.00 (0.99, 1.01)	0.7644	1.01 (1.00, 1.03)	0.0747
sdLDL-C quartile						
Q1	1.0		1.0		1.0	
Q2	0.83 (0.53, 1.31)	0.4208	0.95 (0.60, 1.51)	0.8165	1.15 (0.59, 2.21)	0.6850
Q3	0.83 (0.53, 1.31)	0.4208	0.89 (0.56, 1.42)	0.6219	1.42 (0.76, 2.67)	0.2691
Q4	0.70 (0.44, 1.11)	0.1284	0.87 (0.54, 1.43)	0.5910	1.46 (0.75, 2.87)	0.2660
P for trend	0.1481		0.5503		0.2018	
(h)						
TG/HDL-C	0.89 (0.82, 0.97)	**0.0065**	0.94 (0.86, 1.02)	0.1592	0.96 (0.86, 1.07)	0.4949
TG/HDL-C quartile						
Q1	1.0		1.0		1.0	
Q2	0.80 (0.52, 1.24)	0.3202	0.95 (0.61, 1.49)	0.8222	1.20 (0.65, 2.22)	0.5561
Q3	0.49 (0.31, 0.79)	**0.0029**	0.60 (0.37, 0.97)	**0.0367**	0.85 (0.44, 1.62)	0.6178
Q4	0.46 (0.29, 0.74)	**0.0013**	0.65 (0.40, 1.08)	0.0942	0.88 (0.45, 1.74)	0.7117
P for trend	**0.0002**		**0.0277**		0.4949	
(i)						
TC/HDL-C	0.97 (0.83, 1.13)	0.6823	1.07 (0.91, 1.26)	0.4102	1.27 (1.02, 1.57)	**0.0311**
TC/HDL-C quartile						
Q1	1.0		1.0		1.0	
Q2	1.21 (0.77, 1.91)	0.4152	1.35 (0.85, 2.16)	0.2082	1.64 (0.84, 3.20)	0.1485
Q3	1.15 (0.72, 1.82)	0.5580	1.30 (0.81, 2.09)	0.2773	1.76 (0.90, 3.42)	0.0971
Q4	0.92 (0.57, 1.47)	0.7177	1.22 (0.74, 2.01)	0.4283	2.36 (1.20, 4.65)	**0.0129**
P for trend	0.6830		0.4613		**0.0159**	
(j)						
LDL-C/HDL-C	1.03 (0.87, 1.22)	0.7442	1.14 (0.95, 1.36)	0.1567	1.43 (1.13, 1.81)	**0.0029**
LDL-C/HDL-C quartile						
Q1	1.0		1.0		1.0	
Q2	1.42 (0.90, 2.26)	0.1308	1.70 (1.06, 2.73)	**0.0289**	2.45 (1.23, 4.85)	**0.0105**
Q3	1.15 (0.72, 1.85)	0.5499	1.33 (0.82, 2.16)	0.2455	2.30 (1.17, 4.53)	**0.0162**
Q4	1.09 (0.68, 1.75)	0.7177	1.40 (0.86, 2.29)	0.1789	2.85 (1.43, 5.67)	**0.0028**
P for trend	0.9704		0.3371		**0.0072**	
(k)						
lbLDL-C/HDL-C	1.21 (0.94, 1.56)	0.1337	1.34 (1.03, 1.74)	**0.0286**	1.86 (1.32, 2.63)	**0.0004**
lbLDL-C/HDL-C quartile						
Q1	1.0		1.0		1.0	
Q2	1.35 (0.85, 2.15)	0.1980	1.51 (0.94, 2.43)	0.0886	2.33 (1.20, 4.52)	**0.0124**
Q3	0.86 (0.53, 1.40)	0.5340	0.97 (0.59, 1.60)	0.8969	1.45 (0.71, 2.96)	0.3102
Q4	1.50 (0.95, 2.37)	0.0834	1.81 (1.12, 2.90)	**0.0147**	3.10 (1.60, 6.01)	**0.0008**
P for trend	0.2820		0.0764		**0.0057**	
(l)						
sdLDL-C/HDL-C	0.69 (0.45, 1.07)	0.0980	0.95 (0.60, 1.51)	0.8320	1.54 (0.85, 2.80)	0.1519
sdLDL-C/HDL-C quartile						
Q1	1.0		1.0		1.0	
Q2	1.17 (0.75, 1.81)	0.4983	1.32 (0.84, 2.08)	0.2316	1.39 (0.73, 2.65)	0.3198
Q3	0.73 (0.46, 1.17)	0.1921	0.84 (0.52, 1.35)	0.4654	1.35 (0.70, 2.59)	0.3653
Q4	0.75 (0.47, 1.20)	0.2372	1.07 (0.65, 1.76)	0.7769	1.80 (0.91, 3.58)	0.0912
P for trend	0.0815		0.7316		0.1202	
(m)						
RC/HDL-C	0.59 (0.36, 0.96)	0.0347	0.70 (0.42, 1.16)	0.1694	0.54 (0.27, 1.06)	0.0740
RC/HDL-C quartile						
Q1	1.0		1.0		1.0	
Q2	1.47 (0.94, 2.30)	0.0903	1.50 (0.95, 2.37)	0.0832	1.75 (0.93, 3.29)	0.0807
Q3	0.89 (0.56, 1.43)	0.6311	1.00 (0.62, 1.62)	0.9990	1.32 (0.69, 2.53)	0.3943
Q4	0.84 (0.52, 1.35)	0.4682	1.01 (0.62, 1.65)	0.9701	1.47 (0.78, 2.79)	0.2334
P for trend	0.1700		0.6396		0.4094	
(n)						
NHHR	0.97 (0.83, 1.13)	0.6823	1.07 (0.91, 1.26)	0.4102	1.27 (1.02, 1.57)	**0.0311**
NHHR quartile						
Q1	1.0		1.0		1.0	
Q2	1.21 (0.77, 1.91)	0.4152	1.35 (0.85, 2.16)	0.2082	1.64 (0.84, 3.20)	0.1485
Q3	1.15 (0.72, 1.82)	0.5580	1.30 (0.81, 2.09)	0.2773	1.76 (0.90, 3.42)	0.0971
Q4	0.92 (0.57, 1.47)	0.7177	1.22 (0.74, 2.01)	0.4283	2.36 (1.20, 4.65)	**0.0129**
P for trend	0.6830		0.4613		**0.0159**	

Continuous variables were analyzed per unit increase. Quartile analyses were performed using the lowest quartile (Q1) as the reference category. Model 1 was unadjusted. Model 2 was adjusted for age and sex. Model 3 was further adjusted for smoking status, hypertension, diabetes, atrial fibrillation, TOAST classification, and baseline NIHSS score. P for trend was calculated by entering the median value of each quartile as a continuous variable in the regression model. Bold values indicate statistically significant *p* values (*p* < 0.05). Odds ratios (ORs) and 95% confidence intervals (CIs) were estimated using logistic regression. TC, total cholesterol; TG, triglycerides; HDL-C, high-density lipoprotein cholesterol; LDL-C, low-density lipoprotein cholesterol; lbLDL-C, large buoyant low-density lipoprotein cholesterol; sdLDL-C, small dense low-density lipoprotein cholesterol; RC, remnant cholesterol; NHHR, non–HDL-C to HDL-C ratio; TOAST, Trial of Org 10,172 in Acute Stroke Treatment classification; NIHSS, National Institutes of Health Stroke Scale.

TC, LDL-C, and lbLDL-C were positively associated with risk when analyzed as continuous variables. Patients in the highest quartiles of these parameters had significantly higher odds of poor functional outcome compared with those in the lowest quartiles.

Among lipid ratios, LDL-C/HDL-C, lbLDL-C/HDL-C, TC/HDL-C, and NHHR remained independently associated with outcome. The strongest associations were observed for lbLDL-C/HDL-C and LDL-C/HDL-C. TC/HDL-C and NHHR demonstrated identical effect estimates in the fully adjusted model.

In contrast, TG, HDL-C, RC, sdLDL-C, TG/HDL-C, sdLDL-C/HDL-C, and RC/HDL-C were not significantly associated with outcome.

### Smooth curve analyses using generalized additive models

**Figure 1:**
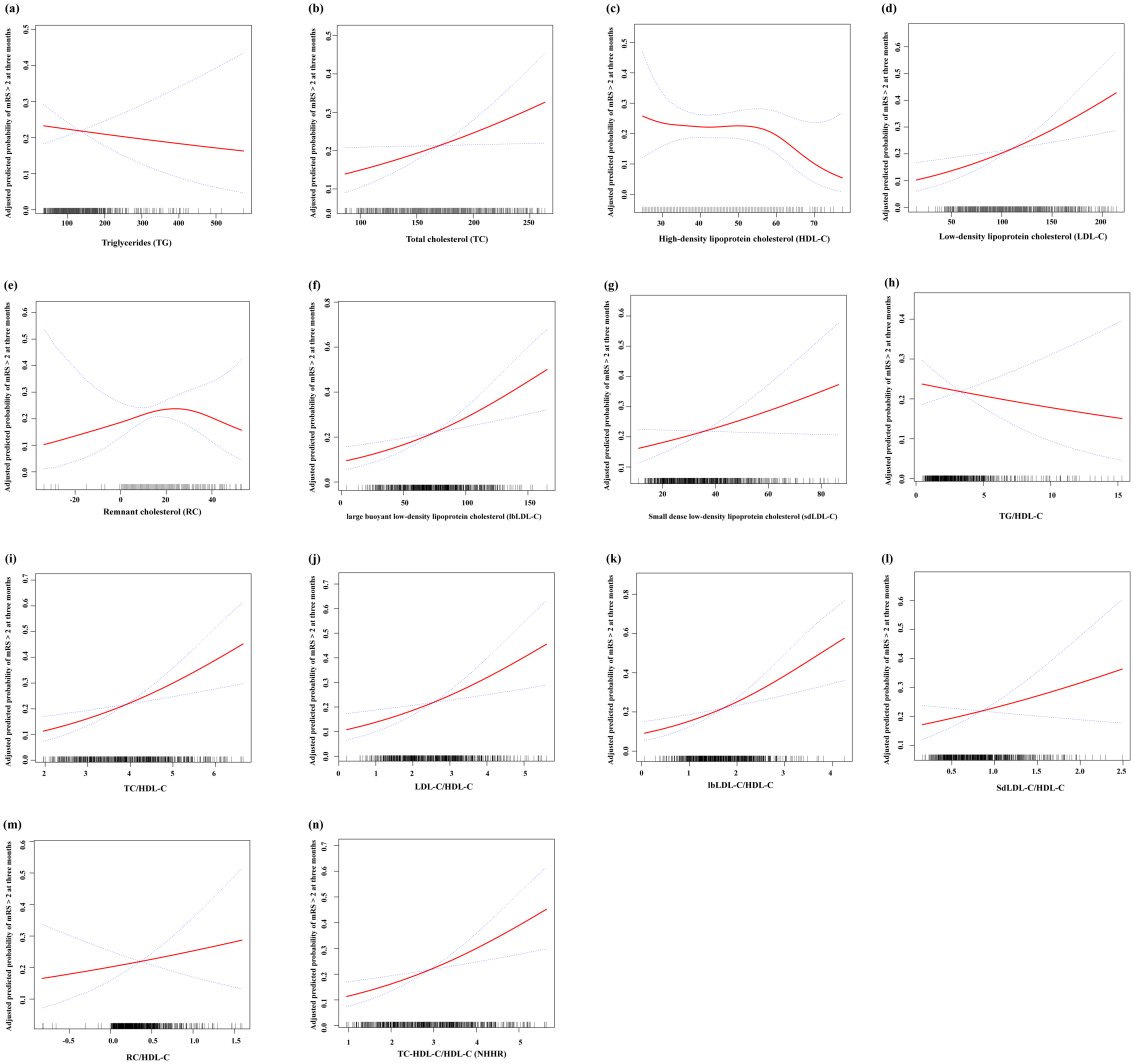
Generalized additive model–based smooth curves showing the associations between lipid-related parameters and 3-month poor functional outcome. Panels **(a–n)** present triglycerides (TG), total cholesterol (TC), high-density lipoprotein cholesterol (HDL-C), low-density lipoprotein cholesterol (LDL-C), remnant cholesterol (RC), large buoyant LDL cholesterol (lbLDL-C), small dense LDL cholesterol (sdLDL-C), TG/HDL-C, TC/HDL-C, LDL-C/HDL-C, lbLDL-C/HDL-C, sdLDL-C/HDL-C, RC/HDL-C, and non–HDL-C to HDL-C ratio (NHHR), respectively. The red lines represent adjusted predicted probabilities derived from fully adjusted logistic models, and the blue dotted lines indicate 95% confidence intervals. Tick marks along the x-axis represent the distribution of observations. Models were adjusted for age, sex, smoking status, hypertension, diabetes, atrial fibrillation, Trial of Org 10,172 in Acute Stroke Treatment (TOAST) classification, and baseline National Institutes of Health Stroke Scale (NIHSS) score.

Fully adjusted generalized additive models (Figure 1) showed decreasing trends for TG and TG/HDL-C across their distributions. In exploratory smooth-curve analyses, HDL-C and RC showed possible non-linear patterns with apparent turning points. HDL-C exhibited a relatively flat mid-range followed by a decline at higher levels, whereas RC displayed an increase at intermediate levels and a subsequent decline.

The remaining lipid parameters generally showed increasing probabilities of poor functional outcome with higher exposure levels.

### Threshold effect analyses of lipid parameters

Two-piecewise logistic regression analyses (Table 3) suggested threshold effects for HDL-C and possible non-linear model fit for RC.

**Table 3:** Threshold effects of lipid parameters on poor functional outcome (three-month mRS > 2).

3-month mRS >2	Adjusted OR (95% CI)	*p* value
TG (Model I)	1.00 (1.00, 1.00)	0.6033
TG (Model II)		
Inflection point	54.91 (51.37, 68.2)	
TG < 54.91	0.93 (0.85, 1.01)	0.0745
TG > = 54.91	1.00 (1.00, 1.00)	0.8881
Log likelihood ratio	0.086	
TC (Model I)	1.01 (1.00, 1.01)	**0.0365**
TC (Model II)		
Inflection point	108.66 (106.34, 129.16)	
TC < 108.66	0.95 (0.87, 1.03)	0.1887
TC > = 108.66	1.01 (1.00, 1.01)	**0.0146**
Log likelihood ratio	0.168	
HDL-C (Model I)	0.99 (0.97, 1.01)	0.2707
HDL-C (Model II)		
Inflection point	60.33 (56.84, 63.42)	
HDL-C < 60.33	1.00 (0.98, 1.03)	0.7569
HDL-C > = 60.33	0.81 (0.67, 0.98)	**0.0265**
Log likelihood ratio	**0.014**	
LDL-C (Model I)	1.01 (1.00, 1.02)	**0.0019**
LDL-C (Model II)		
Inflection point	85.07 (71.15, 93.19)	
LDL-C < 85.07	1.02 (0.99, 1.04)	0.1883
LDL-C > = 85.07	1.01 (1.00, 1.02)	**0.0384**
Log likelihood ratio	0.566	
RC (Model I)	1.01 (0.98, 1.03)	0.6167
RC (Model II)		
Inflection point	32.10 (25.14, 37.9)	
RC < 32.10	1.02 (1.00, 1.05)	0.0892
RC > = 32.10	0.85 (0.70, 1.02)	0.0789
Log likelihood ratio	**0.017**	
lbLDL-C (Model I)	1.01 (1.01, 1.02)	**0.0008**
lbLDL-C (Model II)		
Inflection point	112.5 (95.43, 122.33)	
lbLDL-C < 112.5	1.02 (1.01, 1.03)	**0.0014**
lbLDL-C > = 112.5	1.00 (0.96, 1.04)	0.8656
Log likelihood ratio	0.389	
sdLDL-C (Model I)	1.01 (1.00, 1.03)	0.0747
sdLDL-C (Model II)		
Inflection point	19.87 (16.91, 23.37)	
sdLDL-C < 19.87	0.93 (0.75, 1.15)	0.4908
sdLDL-C > = 19.87	1.02 (1.00, 1.04)	**0.0491**
Log likelihood ratio	0.413	
TG/HDL-C (Model I)	0.96 (0.86, 1.07)	0.4949
TG/HDL-C (Model II)		
Inflection point	1.55 (1.26, 2.01)	
TG/HDL-C < 1.55	1.76 (0.55, 5.65)	0.3438
TG/HDL-C > = 1.55	0.94 (0.84, 1.06)	0.3208
Log likelihood ratio	0.303	
TC/HDL-C (Model I)	1.49 (1.17, 1.90)	**0.0013**
TC/HDL-C (Model II)		
Inflection point	5.34 (4.94, 5.94)	
TC/HDL-C < 5.34	1.36 (1.02, 1.82)	**0.0379**
TC/HDL-C > = 5.34	2.99 (0.87, 10.26)	0.0823
Log likelihood ratio	0.264	
LDL-C/HDL-C (Model I)	1.43 (1.13, 1.81)	**0.0029**
LDL-C/HDL-C (Model II)		
Inflection point	4.22 (3.46, 4.36)	
LDL-C/HDL-C < 4.22	1.51 (1.15, 2.00)	**0.0032**
LDL-C/HDL-C > = 4.22	0.82 (0.19, 3.50)	0.7877
Log likelihood ratio	0.429	
lbLDL-C/HDL-C (Model I)	1.86 (1.32, 2.63)	**0.0004**
lbLDL-C/HDL-C (Model II)		
Inflection point	1.44 (1.23, 1.61)	
lbLDL-C/HDL-C < 1.44	2.61 (0.81, 8.37)	0.1064
lbLDL-C/HDL-C > = 1.44	1.69 (1.05, 2.70)	**0.0304**
Log likelihood ratio	0.546	
sdLDL-C/HDL-C (Model I)	1.54 (0.85, 2.80)	0.1519
sdLDL-C/HDL-C (Model II)		
Inflection point	0.53 (0.44, 0.64)	
sdLDL-C/HDL-C < 0.53	8.68 (0.20, 372.53)	0.2598
sdLDL-C/HDL-C > = 0.53	1.32 (0.66, 2.62)	0.4281
Log likelihood ratio	0.356	
RC/HDL-C (Model I)	1.34 (0.61, 2.95)	0.4678
RC/HDL-C (Model II)		
Inflection point	0.85 (0.58, 1.03)	
RC/HDL-C < 0.85	2.08 (0.76, 5.69)	0.1555
RC/HDL-C > = 0.85	0.12 (0.00, 5.60)	0.2801
Log likelihood ratio	0.148	
NHHR (Model I)	1.49 (1.17, 1.90)	**0.0013**
NHHR (Model II)		
Inflection point	4.34 (3.95, 4.94)	
NHHR < 4.34	1.36 (1.02, 1.82)	**0.0379**
NHHR > = 4.34	2.99 (0.87, 10.26)	0.0823
Log likelihood ratio	0.264	

Threshold effects were evaluated using two-piecewise logistic regression models. Model I represents the fully adjusted logistic regression model. Model II represents the two-piecewise model with the estimated inflection point. The inflection point was determined using a recursive algorithm. Log likelihood ratio tests were used to compare the one-line and two-line models. Odds ratios (ORs) and 95% confidence intervals (CIs) were adjusted for age, sex, smoking status, hypertension, diabetes, atrial fibrillation, TOAST classification, and baseline NIHSS score. *p* < 0.05 was considered statistically significant. Bold values indicate statistically significant *p* values (*p* < 0.05).

For HDL-C, the inflection point was 60.33 mg/dL. No association was observed below this value, whereas levels ≥60.33 mg/dL were associated with reduced risk. The log likelihood ratio test was significant.

For RC, the estimated inflection point was 32.10 mg/dL. However, the segment-specific estimates were not statistically significant. Therefore, this finding should be interpreted as exploratory, although the log-likelihood ratio test suggested that the two-piecewise model fitted the data better than the linear model.

No significant threshold effects were detected for other lipid parameters.

### Sensitivity and incremental-value analyses of core lipid parameters

Sensitivity analyses after excluding extreme outliers demonstrated that the independent prognostic values of both core indices remained robust (Supplementary Table 1). In the fully adjusted models, the continuous lbLDL-C/HDL-C ratio remained significantly associated with poor functional outcome (OR = 1.78, 95% CI: 1.23–2.57, *p* = 0.0021), and the highest quartile continued to show an increased risk (Q4 vs. Q1: OR = 2.87, 95% CI: 1.47–5.60, *p* = 0.0020; P for trend = 0.0095). Similarly, the continuous NHHR metric remained independently associated with poor functional outcome (OR = 1.36, 95% CI: 1.09–1.71, *p* = 0.0075; P for trend = 0.0140), with patients in the highest quartile exhibiting significantly higher odds of poor functional outcome compared with those in the lowest quartile (Q4 vs. Q1: OR = 2.43, 95% CI: 1.23–4.81, *p* = 0.0110). In an additional sensitivity analysis accounting for inflammatory burden, further adjustment for CRP did not materially alter the associations between the principal LDL-related lipid ratios and poor functional outcome (Supplementary Table 2). Specifically, lbLDL-C/HDL-C and LDL-C/HDL-C remained significantly associated with poor 3-month functional outcome in both continuous and quartile-based analyses after CRP adjustment. We further assessed the incremental prognostic value of LDL-related lipid ratios beyond the clinical base model. The addition of lbLDL-C/HDL-C, LDL-C/HDL-C, TC/HDL-C, or NHHR resulted in modest increases in AUC from 0.8194 to 0.8317, 0.8291, 0.8240, and 0.8240, respectively; however, none of these improvements reached statistical significance. Overall NRI and IDI analyses also showed no significant improvement after adding each lipid ratio (Supplementary Table 3).

### Subgroup analyses stratified by clinical characteristics

In subgroup analyses (Table 4), the associations of lbLDL-C/HDL-C and TC/HDL-C with poor functional outcome were consistent across most strata, with no significant interactions for age, sex, smoking, hypertension, diabetes, or TOAST classification.

**Table 4:** Subgroup analyses and interaction tests for lbLDL-C/HDL-C and TC/HDL-C in relation to 3-month poor functional outcome.

3-month mRS	*N*	OR (95% CI)	*p* value	*p* for interaction
lbLDL-C/HDL-C				
Age				0.8463
<70	358	1.72 (0.99, 2.98)	0.0531	
> = 70	390	1.95 (1.22, 3.12)	0.0053	
Sex				0.9484
Female	310	2.07 (1.26, 3.40)	0.0041	
Male	438	1.71 (1.04, 2.82)	0.0343	
Smoking status				0.2338
No	491	1.65 (1.10, 2.48)	0.0164	
Yes	257	2.24 (1.15, 4.39)	0.0184	
Hypertension				0.5084
No	179	1.47 (0.66, 3.30)	0.3444	
Yes	569	1.93 (1.30, 2.86)	0.0010	
Diabetes				0.3157
No	487	2.09 (1.31, 3.34)	0.0020	
Yes	261	1.70 (1.00, 2.89)	0.0486	
Atrial fibrillation				**0.0078**
No	616	2.32 (1.53, 3.51)	<0.0001	
Yes	132	0.75 (0.35, 1.59)	0.4505	
TOAST				0.1380
LAA	336	2.44 (1.44, 4.13)	0.0009	
Non-LAA	323	1.49 (0.91, 2.43)	0.1104	
TC/HDL-C				
Age				0.8986
<70	358	1.19 (0.87, 1.62)	0.2814	
> = 70	390	1.22 (0.89, 1.66)	0.2173	
Sex				0.7551
Female	310	1.23 (0.89, 1.70)	0.2209	
Male	438	1.31 (0.97, 1.76)	0.0737	
Smoking status				0.5624
No	491	1.18 (0.91, 1.54)	0.2123	
Yes	257	1.31 (0.89, 1.94)	0.1665	
Hypertension				0.5518
No	179	1.08 (0.67, 1.75)	0.7429	
Yes	569	1.28 (1.00, 1.64)	0.0473	
Diabetes				0.2832
No	487	1.47 (1.08, 2.00)	0.0152	
Yes	261	1.15 (0.84, 1.57)	0.3892	
Atrial fibrillation				**0.0036**
No	616	1.45 (1.13, 1.86)	0.0036	
Yes	132	0.67 (0.42, 1.06)	0.0864	
TOAST				0.0646
LAA	336	1.52 (1.11, 2.09)	0.0091	
Non-LAA	323	1.01 (0.73, 1.40)	0.9424	

Odds ratios (ORs) with 95% confidence intervals (CIs) were estimated using multivariable logistic regression models. The fully adjusted model included age, sex, smoking status, hypertension, diabetes, atrial fibrillation (AF), Trial of Org 10,172 in Acute Stroke Treatment (TOAST) classification, and baseline National Institutes of Health Stroke Scale (NIHSS) score. P for interaction was calculated by including a multiplicative interaction term between the lipid ratio and each subgroup variable in the fully adjusted model. Values are presented as OR (95% CI). A two-sided *p* value < 0.05 was considered statistically significant. Bold values indicate statistically significant *p* values (*p* < 0.05).

Atrial fibrillation significantly modified both associations. The lipid ratios were positively associated with poor functional outcome among patients without atrial fibrillation but not among those with atrial fibrillation.

## Discussion

In this retrospective cohort study, the principal finding was that LDL-related lipid ratios, particularly lbLDL-C/HDL-C and LDL-C/HDL-C, were independently associated with poor 3-month functional outcome after acute ischemic stroke. Notably, lbLDL-C/HDL-C showed the strongest signal, with each unit increase associated with an 86% increased risk of poor functional outcome (OR 1.86, 95% CI 1.32–2.63, *p* < 0.001). The main novel contribution of this study is the systematic evaluation of conventional lipid concentrations, calculated lipid parameters, ratio-based indices, and calculated LDL particle-related metrics within a unified analytical framework. These ratio-based indices demonstrated stronger associations with functional outcome compared to isolated lipid concentrations, likely reflecting the critical balance between atherogenic burden and protective functions (10). However, additional AUC, NRI, and IDI analyses showed only modest and statistically non-significant improvement in model discrimination after adding these ratios to the clinical base model. Therefore, these lipid ratios should be interpreted as associative prognostic markers rather than established standalone prediction tools (11).

Previous work has focused largely on conventional, single lipid concentrations like LDL-C in relation to vascular risk and stroke recurrence (12, 13), yielding heterogeneous findings regarding functional recovery. This inconsistency arises because single lipid measures inadequately represent the interplay between atherogenic exposure and protective lipoprotein activity (14). In contrast, our study suggests that ratio-based and particle-related metrics may capture prognostically relevant information not fully reflected by isolated lipid concentrations. By evaluating non-linear trajectories and clinical interaction effects, our findings provide a more nuanced and comprehensive view of how lipid profiles modulate post-stroke recovery.

Atherogenic dyslipidemia alters lipoprotein composition and may be linked to vascular and inflammatory pathways involved in post-stroke repair (15, 16). Ratios incorporating both LDL and HDL components better capture the systemic atherogenic burden and “residual cardiovascular risk” that persists beyond conventional LDL-C targets (17). Following cerebral ischemia, the neurovascular unit undergoes energy failure (18). While small dense LDL particles exacerbate oxidative stress and endothelial damage (19), the non-linear patterns observed for HDL-C and RC suggest a complex metabolic response (20).

Several secondary findings should be interpreted as exploratory. In contrast to LDL-related ratios, RC was not independently associated with functional outcome in the primary adjusted analyses (21–23). The observed non-linear pattern for RC may indicate heterogeneity in the relationship between triglyceride-rich lipoprotein remnants and post-stroke recovery, but this result should be considered exploratory. Further studies with direct remnant lipoprotein measurements and longitudinal metabolic data are needed to clarify whether RC has a clinically meaningful role in stroke recovery (24, 25). Similarly, the inverse association observed for TG and TG/HDL-C aligns with the “Triglyceride Paradox,” where low triglyceride levels often reflect malnutrition or a hypermetabolic state, correlating with severe neurological deficits (26).

The modifying effect of AF should be interpreted cautiously (27). In cardioembolic stroke, thromboembolic mechanisms may play a more prominent role than atherosclerotic pathways (28). The attenuated lipid signals observed in AF patients may reflect differences in dominant stroke mechanisms rather than a direct biological interaction (29, 30). However, this explanation remains speculative because our data did not directly assess thromboembolic burden, atrial remodeling, plaque characteristics, or longitudinal lipid changes. Thus, the AF interaction should be regarded as exploratory and hypothesis-generating. Further studies are needed to determine whether lipid ratios have different prognostic relevance across stroke mechanisms (31, 32).

From a clinical perspective, lipid ratios derived from routine laboratory data may help characterize baseline metabolic risk after stroke (33). Although current guidelines mandate high-intensity statin therapy regardless of baseline lipid levels, our findings suggest that composite lipid indicators may better capture lipoprotein imbalance-related prognostic risk than single parameters (34, 35).

Specifically, ratios like lbLDL-C/HDL-C may help identify patients with lipoprotein imbalance-related prognostic risk based on baseline lipid profiles, although their incremental predictive value beyond established clinical factors appears limited in the present analysis. These findings, however, should be interpreted cautiously; future longitudinal assessments accounting for statin exposure are required to determine if dynamic shifts in lipoprotein balance parallel clinical recovery trajectories (36–38).

### Strengths and limitations

This study benefits from the simultaneous evaluation of multiple lipid measures within a unified analytical framework, exploring non-linearity, threshold effects, and clinical interactions. Crucially, although baseline stroke severity (NIHSS score) differed significantly between the outcome groups, the independent prognostic value of lipoprotein ratios remained intact after robust multivariable adjustment. However, additional AUC, NRI, and IDI analyses showed that their incremental improvement in discrimination beyond the clinical base model was modest and not statistically significant.

Several limitations warrant acknowledgment. The single-center observational design limits generalizability and prevents causal inference. Information on pre-stroke statin use and in-hospital lipid-lowering therapy, including treatment intensity, timing, and adherence, was not systematically available in the retrospective database. Therefore, residual confounding related to lipid-lowering treatment cannot be fully excluded. Although CRP differed significantly between outcome groups, the main multivariable models did not initially include CRP. We therefore performed an additional CRP-adjusted sensitivity analysis, but residual confounding by other inflammatory or acute-phase factors cannot be fully excluded. Furthermore, although high-intensity statin therapy is routinely initiated for acute ischemic stroke patients regardless of their baseline lipid profiles, our findings on composite indicators—such as the lbLDL-C/HDL-C ratio—highlight their value in identifying lipoprotein imbalance-related prognostic risk. These ratios do not challenge standard protocols but may serve as associative prognostic markers for unfavorable 3-month functional outcomes based on their baseline lipoprotein profiles. Lipids were assessed only at baseline, lacking longitudinal dynamics. Additionally, some parameters were calculated rather than directly measured, and apolipoprotein data were unavailable. Despite these constraints, the consistent signals observed for lipoprotein ratios support further investigation into metabolic influences on stroke recovery.

## Conclusion

In patients with acute ischemic stroke, lipoprotein imbalance, particularly reflected by LDL-related ratios, is independently associated with 3-month functional outcome. These associations are heterogeneous across lipid measures and may differ by atrial fibrillation status. This interaction finding should be considered exploratory and requires further validation.

## Data Availability

The raw data supporting the conclusions of this article will be made available by the authors, without undue reservation.
