# Supplementary material for: Association of lipoprotein indices with 3-month functional outcome after ischemic stroke: a retrospective cohort study
Source: Front Nutr. 2026 Aug 3;13:1826387. doi: 10.3389/fnut.2026.1826387 (PMC13478147; doi:10.3389/fnut.2026.1826387)
Supplement: Supplementary file 1 [file Table_1.DOCX]

Supplementary Table 1. Sensitivity Analysis of the Associations of lbLDL-C/HDL-C Ratio and NHHR with 3-Month Functional Outcomes After Outlier Exclusion

(a)

| Exposure | Crude Model (Model 1) | | Partially Adjusted Model  (Model 2) | | Fully Adjusted Model  (Model 3) | |
| --- | --- | --- | --- | --- | --- | --- |
|  | OR (95% CI) | *P*-value | OR (95% CI) | *P*-value | OR (95% CI) | *P*-value |
| lbLDL-C/HDL-C | 1.21 (0.93, 1.58) | 0.1628 | 1.35 (1.03, 1.78) | **0.0321** | 1.78 (1.23, 2.57) | **0.0021** |
| lbLDL-C/HDL-C quartile |  |  |  |  |  |  |
| Q1 | 1.0 |  | 1.0 |  | 1.0 |  |
| Q2 | 1.30 (0.81, 2.06) | 0.2744 | 1.46 (0.90, 2.36) | 0.1211 | 2.24 (1.15, 4.40) | **0.0184** |
| Q3 | 0.88 (0.54, 1.44) | 0.6200 | 1.00 (0.61, 1.65) | 0.9974 | 1.58 (0.78, 3.22) | 0.2036 |
| Q4 | 1.46 (0.92, 2.31) | 0.1046 | 1.77 (1.10, 2.84) | **0.0195** | 2.87 (1.47, 5.60) | **0.0020** |
| P for trend | 0.2844 | | 0.0787 | | **0.0095** | |

(b)

| Exposure | Crude Model (Model 1) | | Partially Adjusted Model  (Model 2) | | Fully Adjusted Model  (Model 3) | |
| --- | --- | --- | --- | --- | --- | --- |
|  | OR (95% CI) | *P*-value | OR (95% CI) | *P*-value | OR (95% CI) | *P*-value |
| NHHR | 0.97 (0.83, 1.14) | 0.6884 | 1.08 (0.91, 1.28) | 0.3810 | 1.36 (1.09, 1.71) | **0.0075** |
| NHHR quartile |  |  |  |  |  |  |
| Q1 | 1.0 |  | 1.0 |  | 1.0 |  |
| Q2 | 1.21 (0.77, 1.91) | 0.4148 | 1.35 (0.84, 2.16) | 0.2142 | 1.66 (0.84, 3.26) | 0.1433 |
| Q3 | 1.12 (0.70, 1.77) | 0.6379 | 1.28 (0.79, 2.06) | 0.3146 | 1.76 (0.90, 3.48) | 0.1010 |
| Q4 | 0.92 (0.57, 1.47) | 0.7175 | 1.22 (0.74, 2.00) | 0.4412 | 2.42 (1.23, 4.81) | **0.0110** |
| P for trend | 0.6552 | | 0.4894 | | **0.0140** | |

Data are presented as odds ratios (ORs) and 95% confidence intervals (CIs) estimated via multivariable logistic regression. Sensitivity analyses were performed after excluding extreme statistical outliers, which were defined as values exceeding 3 standard deviations from the cohort mean. Continuous variables were analyzed per unit increase. Quartile analyses were performed using the lowest quartile (Q1) as the reference category.

Model 1 was unadjusted. Model 2 was adjusted for age and sex. Model 3 was further adjusted for smoking status, hypertension, diabetes, atrial fibrillation, TOAST classification, and baseline NIHSS score. P for trend was calculated by entering the median value of each quartile as a continuous variable in the regression model.

Abbreviations: NHHR, non–high-density lipoprotein cholesterol to high-density lipoprotein cholesterol ratio; lbLDL-C, large buoyant low-density lipoprotein cholesterol; HDL-C, high-density lipoprotein cholesterol; TOAST, Trial of Org 10172 in Acute Stroke Treatment classification; NIHSS, National Institutes of Health Stroke Scale.
