# Supplementary material for: Association of lipoprotein indices with 3-month functional outcome after ischemic stroke: a retrospective cohort study
Source: Front Nutr. 2026 Aug 3;13:1826387. doi: 10.3389/fnut.2026.1826387 (PMC13478147; doi:10.3389/fnut.2026.1826387)
Supplement: Supplementary file 2 [file Table_2.DOCX]

**Supplementary Table 2. Sensitivity analysis of the associations between LDL-related lipid ratios and 3-month functional outcome after additional adjustment for C-reactive protein.**

**(a)**

| Exposure | Crude Model (Model 1) | | Partially Adjusted Model  (Model 2) | | Fully Adjusted Model  (Model 3) | |
| --- | --- | --- | --- | --- | --- | --- |
|  | OR (95% CI) | *P value* | OR (95% CI) | *P value* | OR (95% CI) | *P value* |
| lbLDL-C/HDL-C | 1.21 (0.94, 1.56) | 0.1337 | 1.34 (1.03, 1.74) | **0.0286** | 1.86 (1.31, 2.62) | **0.0005** |
| lbLDL-C/HDL-C quartile |  |  |  |  |  |  |
| Q1 | 1.0 |  | 1.0 |  | 1.0 |  |
| Q2 | 1.35 (0.85, 2.15) | 0.1980 | 1.51 (0.94, 2.43) | 0.0886 | 2.32 (1.20, 4.50) | **0.0128** |
| Q3 | 0.86 (0.53, 1.40) | 0.5340 | 0.97 (0.59, 1.60) | 0.8969 | 1.44 (0.71, 2.95) | 0.3145 |
| Q4 | 1.50 (0.95, 2.37) | 0.0834 | 1.81 (1.12, 2.90) | **0.0147** | 3.10 (1.60, 6.01) | **0.0008** |
| P for trend | 0.2820 | | 0.0764 | | **0.0057** | |

**(b)**

| Exposure | Crude Model (Model 1) | | Partially Adjusted Model  (Model 2) | | Fully Adjusted Model  (Model 3) | |
| --- | --- | --- | --- | --- | --- | --- |
|  | OR (95% CI) | *P value* | OR (95% CI) | *P value* | OR (95% CI) | *P value* |
| LDL-C/HDL-C | 1.03 (0.87, 1.22) | 0.7442 | 1.14 (0.95, 1.36) | 0.1567 | 1.43 (1.13, 1.81) | **0.0030** |
| LDL-C/HDL-C quartile |  |  |  |  |  |  |
| Q1 | 1.0 |  | 1.0 |  | 1.0 |  |
| Q2 | 1.42 (0.90, 2.26) | 0.1308 | 1.70 (1.06, 2.73) | **0.0289** | 2.45 (1.23, 4.85) | **0.0105** |
| Q3 | 1.15 (0.72, 1.85) | 0.5499 | 1.33 (0.82, 2.16) | 0.2455 | 2.29 (1.16, 4.52) | **0.0166** |
| Q4 | 1.09 (0.68, 1.75) | 0.7177 | 1.40 (0.86, 2.29) | 0.1789 | 2.85 (1.44, 5.67) | **0.0028** |
| P for trend | 0.9704 | | 0.3371 | | **0.0072** | |

**(c)**

| Exposure | Crude Model (Model 1) | | Partially Adjusted Model  (Model 2) | | Fully Adjusted Model  (Model 3) | |
| --- | --- | --- | --- | --- | --- | --- |
|  | OR (95% CI) | *P value* | OR (95% CI) | *P value* | OR (95% CI) | *P value* |
| TC/HDL-C | 0.97 (0.83, 1.13) | 0.6823 | 1.07 (0.91, 1.26) | 0.4102 | 1.27 (1.02, 1.57) | **0.0315** |
| TC/HDL-C quartile |  |  |  |  |  |  |
| Q1 | 1.0 |  | 1.0 |  | 1.0 |  |
| Q2 | 1.21 (0.77, 1.91) | 0.4152 | 1.35 (0.85, 2.16) | 0.2082 | 1.63 (0.84, 3.19) | 0.1504 |
| Q3 | 1.15 (0.72, 1.82) | 0.5580 | 1.30 (0.81, 2.09) | 0.2773 | 1.76 (0.90, 3.42) | 0.0969 |
| Q4 | 0.92 (0.57, 1.47) | 0.7177 | 1.22 (0.74, 2.01) | 0.4283 | 2.36 (1.20, 4.64) | **0.0133** |
| P for trend | 0.6830 | | 0.4613 | | **0.0162** | |

**(d)**

| Exposure | Crude Model (Model 1) | | Partially Adjusted Model  (Model 2) | | Fully Adjusted Model  (Model 3) | |
| --- | --- | --- | --- | --- | --- | --- |
|  | OR (95% CI) | *P value* | OR (95% CI) | *P value* | OR (95% CI) | *P value* |
| NHHR | 0.97 (0.83, 1.13) | 0.6823 | 1.07 (0.91, 1.26) | 0.4102 | 1.27 (1.02, 1.57) | **0.0315** |
| NHHR quartile |  |  |  |  |  |  |
| Q1 | 1.0 |  | 1.0 |  | 1.0 |  |
| Q2 | 1.21 (0.77, 1.91) | 0.4152 | 1.35 (0.85, 2.16) | 0.2082 | 1.63 (0.84, 3.19) | 0.1504 |
| Q3 | 1.15 (0.72, 1.82) | 0.5580 | 1.30 (0.81, 2.09) | 0.2773 | 1.76 (0.90, 3.42) | 0.0969 |
| Q4 | 0.92 (0.57, 1.47) | 0.7177 | 1.22 (0.74, 2.01) | 0.4283 | 2.36 (1.20, 4.64) | **0.0133** |
| P for trend | 0.6830 | | 0.4613 | | **0.0162** | |

Note: Continuous variables were analyzed per unit increase. Quartile analyses were performed using the lowest quartile (Q1) as the reference category. Model 1 was unadjusted. Model 2 was adjusted for age and sex. Model 3 was further adjusted for smoking status, hypertension, diabetes, atrial fibrillation, TOAST classification, baseline NIHSS score, and C-reactive protein. P for trend was calculated by entering the median value of each quartile as a continuous variable in the regression model. Odds ratios (ORs) and 95% confidence intervals (CIs) were estimated using logistic regression.

Abbreviations: TC, total cholesterol; HDL-C, high-density lipoprotein cholesterol; LDL-C, low-density lipoprotein cholesterol; lbLDL-C, large buoyant low-density lipoprotein cholesterol; NHHR, non-HDL-C to HDL-C ratio; CRP, C-reactive protein; TOAST, Trial of Org 10172 in Acute Stroke Treatment classification; NIHSS, National Institutes of Health Stroke Scale.
