# Supplementary material for: Association of lipoprotein indices with 3-month functional outcome after ischemic stroke: a retrospective cohort study
Source: Front Nutr. 2026 Aug 3;13:1826387. doi: 10.3389/fnut.2026.1826387 (PMC13478147; doi:10.3389/fnut.2026.1826387)
Supplement: Supplementary file 3 [file Table_3.DOCX]

**Supplementary Table 3. Incremental prognostic value of LDL-related lipid ratios beyond the clinical base model**

| **Model** | **AUC** | **95% CI** | **ΔAUC** | **P for AUC comparison** | **NRI** | **P for NRI** | **IDI** | **P for IDI** |
| --- | --- | --- | --- | --- | --- | --- | --- | --- |
| Clinical base model | 0.8194 | 0.7843–0.8632 | Reference | Reference | Reference | Reference | Reference | Reference |
| Clinical base model + lbLDL-C/HDL-C | 0.8317 | 0.7960–0.8758 | 0.0123 | 0.1355 | 0.0391 | 0.1581 | 0.0391 | 0.1592 |
| Clinical base model + LDL-C/HDL-C | 0.8291 | 0.7908–0.8663 | 0.0097 | 0.1698 | 0.0407 | 0.1573 | 0.0407 | 0.1585 |
| Clinical base model + TC/HDL-C | 0.8240 | 0.7874–0.8646 | 0.0046 | 0.4221 | 0.0362 | 0.2580 | 0.0362 | 0.2593 |
| Clinical base model + NHHR | 0.8240 | 0.7880–0.8704 | 0.0046 | 0.3999 | 0.0362 | 0.2580 | 0.0362 | 0.2593 |

**Note:** The clinical base model included age, sex, smoking status, hypertension, diabetes, atrial fibrillation, TOAST classification, and baseline NIHSS score. Each extended model was constructed by adding one LDL-related lipid ratio to the clinical base model. AUC confidence intervals and P values for AUC comparison were estimated using nonparametric bootstrap resampling with 500 repetitions. NRI was calculated based on the optimal threshold defined by the maximum sum of sensitivity and specificity. IDI was calculated to evaluate overall discrimination improvement after adding each lipid ratio. NHHR is mathematically related to TC/HDL-C because NHHR = TC/HDL-C − 1.

**Abbreviations:** AUC, area under the receiver operating characteristic curve; CI, confidence interval; NRI, net reclassification improvement; IDI, integrated discrimination improvement; TC, total cholesterol; HDL-C, high-density lipoprotein cholesterol; LDL-C, low-density lipoprotein cholesterol; lbLDL-C, large buoyant low-density lipoprotein cholesterol; NHHR, non-HDL-C to HDL-C ratio; TOAST, Trial of Org 10172 in Acute Stroke Treatment classification; NIHSS, National Institutes of Health Stroke Scale.
